# Depression among keratoconus patients: a systematic review and meta-analysis

**DOI:** 10.3389/fpubh.2024.1477411

**Published:** 2024-11-22

**Authors:** Reza Moshfeghinia, Ali Arman, Navid Sobhi, Golnoush Mahmoudinezhad, Hossein Molavi Vardanjani

**Affiliations:** ^1^Student Research Committee, Shiraz University of Medical Sciences, Shiraz, Iran; ^2^MD-MPH Department, School of Medicine, Shiraz University of Medical Sciences, Shiraz, Iran; ^3^Research Center for Psychiatry and Behavioral Sciences, Shiraz University of Medical Sciences, Shiraz, Iran; ^4^Nikookari Eye Center, Tabriz University of Medical Sciences, Tabriz, Iran; ^5^Department of Ophthalmology at the Shiley Eye Institute, University of California at San Diego, La Jolla, CA, United States

**Keywords:** keratoconus, depression, mental health, systematic review, meta-analysis

## Abstract

**Background:**

Keratoconus (KC) is a chronic corneal disease that typically presents in early adulthood, and may potentially result in poor mental health in affected individuals. The evidence regarding the association of depression with KC is controversial. Hence, we investigated the association between depression and KC via a systematic review and meta-analysis.

**Methods:**

Five electronic medical databases (PubMed, Scopus, PsycINFO, Web of Science, and CINAHL Complete) were systematically queried for English-language records from their inception to January 8, 2024. We include observational studies that measured the risk of depression or compared depression scores in KC patients in comparison to healthy ones. The Newcastle–Ottawa Quality Assessment Scale was utilized to assess bias risk in the included studies. Random-effect modeling was applied for meta-analysis (STATA-17).

**Results:**

Out of the 159 documents retrieved, seven articles were deemed relevant after screening. An analysis involving 83 KC patients and 3,186 controls indicated that KC participants had significantly higher depression scores [SMD: 0.71 [0.31, 1.11]; *p* < 0.01, *I*^2^: 52.7%]. However, a meta-analysis of four studies comparing depression rates in KC patients (*n* = 23,838) to control groups (*n* = 73,482) found no increased risk of depression among KC patients compared to controls [OR: 1.13 [0.66, 1.94]; *p* = 0.65, *I*^2^: 95.35%].

**Conclusion:**

While KC patients exhibit significantly higher depression scores compared to controls, a meta-analysis indicates no increased overall risk of depression among KC patients. These findings highlight the complexity of the relationship between keratoconus and mental health, warranting further investigation.

**Systematic review registration:**

PROSPERO, identifier, CRD42024502247, available at: https://www.crd.york.ac.uk/prospero/display_record.php?ID=CRD42024502247.

## 1 Introduction

Keratoconus (KC) is a bilateral chronic corneal disease characterized by progressive thinning and steeping of the cornea that changes the normal dome shape of the cornea into a cone-shaped one. This change results in irregular astigmatism and myopia (1). The global prevalence of KC has been reported as 1.38 per 1,000 population (2). However, the Asian population, particularly the Middle Eastern countries, is at significantly higher risk of developing KC (3, 4). The incidence of KC is typically higher among young adults in the third decade of life (5, 6).

Although KC is rare, its chronic nature and typical onset in young adulthood have raised a concern about its potential psychological impact (7). Adolescence and young adulthood are key stages in which individuals undergo physical and psychosocial changes, seek their goals, and shape their future (8). Hence, a chronic progressive eye disorder in early adulthood, which can lead to significant vision loss, may profoundly affect patients in terms of psychosocial problems, quality of life, and treatment adherence (9, 10).

Studies reported higher psychiatric disorders and lower quality of life among KC patients (11, 12). In a study in Saudi Arabia, the prevalence of anxiety and depression was 63.2 and 56.1% respectively among KC patients (12). Also, in a study in Turkey, the rate of psychiatric diagnosis and moderate-severe depression was 37.2 and 13.8%, respectively (11). However, studies assessing depression in KC patients compared to control have yielded conflicting results. Lin et al. (13) in a population-based study, reported depression as a protective factor against developing KC. In contrast, some studies suggest a significant association between KC and depression (14, 15), while other studies have reported no significant association (16, 17).

Individuals suffering from depression are at greater risk of life-threatening situations like suicide and tend to have a lower quality of life (18, 19). So, the potential link between depression and KC has raised concern about the potential psychological consequences of KC.

This systematic review and meta-analysis aim to comprehensively assess the prevalence and risk of depression among individuals diagnosed with KC compared to healthy controls.

## 2 Methods

This systematic review and meta-analysis followed the Preferred Reporting Items for Systematic Reviews and Meta-Analyses (PRISMA) guidelines 2020 (20). The registration number in PROSPERO is CRD42024502247. The PRISMA checklist is included in Supplementary material 1.

### 2.1 Search strategy

Five electronic databases (PubMed (Medline), PsycINFO, Scopus, Web of Science, and CINAHL Complete) were systematically searched for English-language records from their inception to January 8, 2024. The searches included keyword combinations such as “Keratoconus” AND “Depression” (Supplementary material 2). Additionally, the references of the included studies and Google Scholar were screened to identify potentially eligible articles.

### 2.2 Eligibility criteria

We included observational studies to evaluate the risk of depression and compare depression scores between KC patients and healthy individuals. Our inclusion criteria, based on the PECO framework (Population, Exposure, Comparison, Outcomes), were as follows:

Population and Exposure: Confirmed KC patients.

Comparison: General population.

Outcomes: Depression scores (measured using validated tools) or prevalence of depression.

We excluded studies that met any of the following criteria: (1) Replication of secondary data from other studies, (2) Studies classified as reviews, editorials, conference papers, case series/reports, secondary analyses, or animal studies, and (3) Studies utilizing qualitative research methods.

### 2.3 Study selection

Two authors (AA and RM), independently reviewed the titles and abstracts of potentially eligible studies using Rayyan (21). For studies that seem potentially eligible, authors independently assessed the full texts. Any conflicts concerning study design or methods, as well as the ultimate decision on whether to include studies, were resolved through a consensus meeting with the senior author (HM).

### 2.4 Data extraction

Two authors (AA and RM), independently extracted information from the included articles and all discrepancies were resolved through additional discussions. The following general characteristics were gathered: author name and publication year, study location, study design, sample size, residence of participants including urban and rural, ethnicity, male-to-female ratio, depression tool, the time which has passed from KC diagnosis, primary findings of the included studies, and risk of bias (Table 1).

**Table 1:** General characteristics of all included studies.

**References**	**Country**	**Design**	**Participants (KC, control)**	**Male/ female**	**Age (SD)**	**Urban/rural**	**Depression tool**	**Time from KC diagnosis; year (SD)**	**Findings**	**Limitation**	**Risk of bias**
Xu et al. (23)	China	Population-based cross-sectional study	27; 3,139	4/23, 1,370/1,769	64.2 (11.3), 64.2 (9.7)	7/20, 1,665/1,474	Interview with standardized questions	N/A	Positive significant association in univariate analysis; non-significant association in multivariate analysis.	Unclear definition of depression	Low risk of bias
Woodward et al. (16)	US	Population-based study- case-control study	16,053; 16,053	9,456/6,597; 9,456/6,597	40.4 (13.0); 40.4 (13.0)	14,660/1,263; 14,213/1,730	Data registration	4.7 (2.9)	Non-significant association	Only individuals with health insurance included	Low risk of bias
Moschos et al. (14)	Greece	Case-control study	56; 47	34/22; 30/17	41 (7); 42 (9)	N/A	Zung SDS-PHQ9	N/A	Positive significant association	Unknown duration of KC	Low risk of bias
Bak-Nielsen et al. (22)	Denmark	Nationwide population-based study- case-control study	2,679; 26,790	1,791/888; 17,910/8,880	38.2 (15.9); 38.2 (15.9)	N/A	Data Registration	N/A	Positive significant association after considering the time from diagnosis (108% higher odds of depression compared to controls)	The duration of KC was not precisely clarified; although it is considered in the analysis	Low risk of bias
Lin et al. (13)	Taiwan	Nationwide population-based study- case-control study	5,055; 20,220	2,991/2,064; 11,964/8,256	29.76 (12.02); 29.76 (12.02)	1,717/1,566; 5,846/7,538	Data registration	9.85 (4.75)	Negative significant association	Diagnosis of KC was not confirmed with health records	Low risk of bias
Aslan et al. (15)	Turkey	Case-control study	59; 65	41/18; 40/25	23.98 (5.7); 25.82 (5.4)	N/A	BDI-21	N/A^*^1	Positive significant association	Being the majority of KC patients in the early stages	Low risk of bias
Marx-Gross et al. (17)	German	Prospective population-based cohort study	51; 10,368	28/23; 5,352/5,067	40–80^*^2	1	PHQ-9	N/A^*3^	Non-significant association	Unknown duration of KC; Exclusion of mentally ill persons who were unable to participate in the study	Low risk of bias

^*^1Majority of KC patients were in Stages I and II (relatively earlier stages).

^*^2All participants' age span.

^*^3All participants are above 40 years old at baseline; Thus, it can be assumed that all subjects with keratoconus had already developed the disease at the start of the cohort.

KC, keratoconus; BDI-21, Beck Depression Inventory-21; PHQ-9, Patient Health Questionnaire-9; N/A, Not Available; SDS, Zung Self-Rating Depression Scale; CDVA, Corrected distance visual acuity; CXL, corneal cross-linking.

### 2.5 Quality assessment

We utilized the Newcastle–Ottawa Quality Assessment Scale to assess bias risk in the cohort, case-control, and cross-sectional studies included in our analysis. Studies were categorized as having either a low (≥7 stars), moderate (5–6 stars), or high risk of bias (≤ 4 stars), with an overall quality score of nine stars.

### 2.6 Quantitative analysis

We use two types of data for analysis. First, we use, standard mean differences (SMDs) were employed to account for variations in depression measurement methods across diverse studies. In our research, we utilized SMDs along with a 95% confidence interval (CI) to evaluate the disparities in depression scores between KC and control groups. Second, to obtain the comparison of the risk of depression between KC and control groups, odds ratio (OR) and standard error (SE) statistics were used. A random effects model (restricted maximum–likelihood model) was used to pool the extracted OR. Heterogeneity between the studies was evaluated by using the chi-square test and I square statistic. Publication bias was assessed by using the Begg and Egger tests. A meta-regression analysis was conducted to assess the impact of the publication year, total population, average age, and percentage of males. A sensitivity analysis was also carried out to test the robustness of the pooled effect size. All analyses were performed in Stata software (version 17, Stata Corporation, College Station, Texas, USA). *P*-values < 0.05 were considered statistically significant.

## 3 Results

### 3.1 Selection of studies

**Figure 1:**
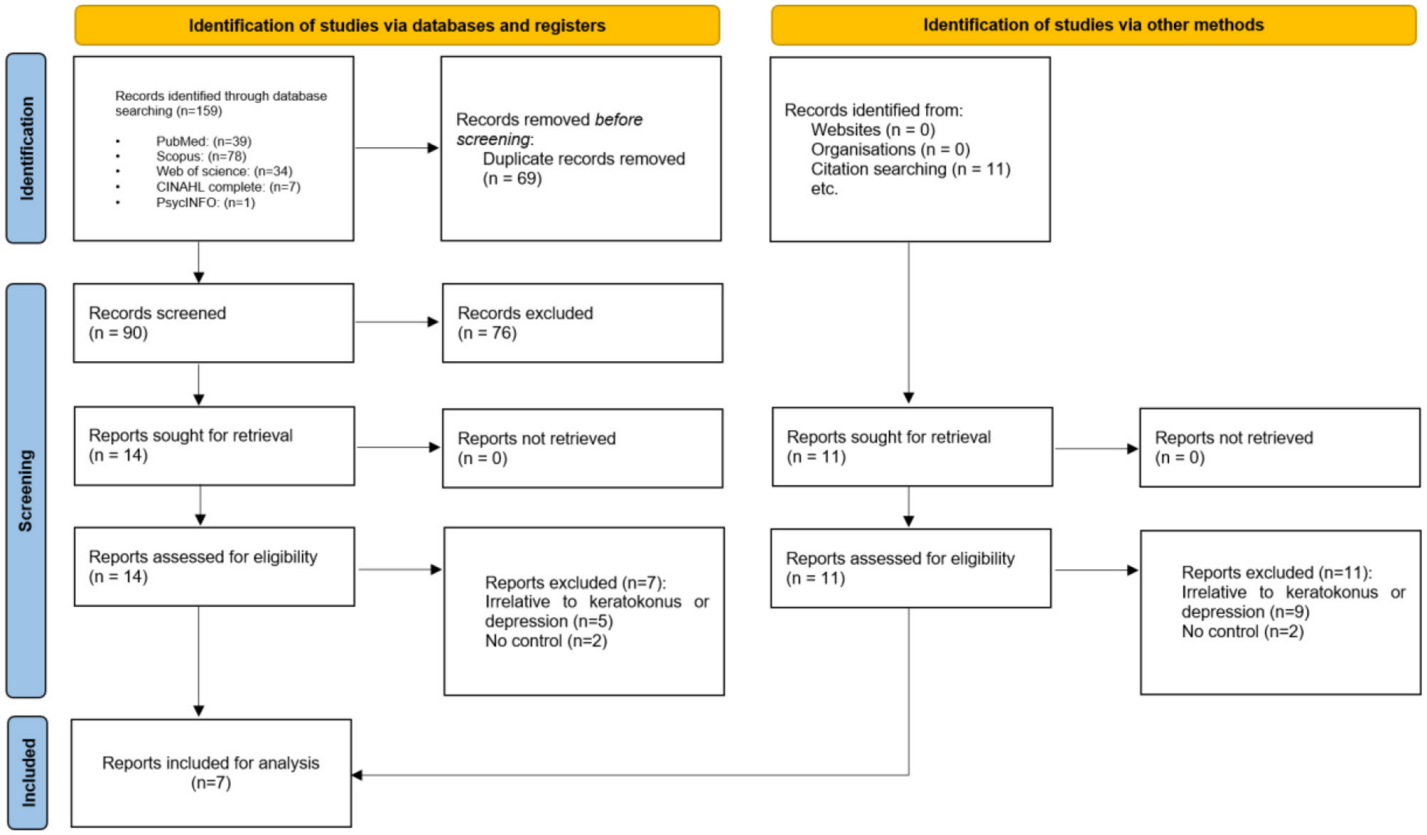
PRISMA of all included studies.

Initially, the search criteria generated 159 articles. After eliminating 69 duplicates using EndNote, we excluded 90 articles following title and abstract screening. Subsequently, based on the eligibility criteria, we identified 14 articles as potentially relevant to our systematic review. Following a thorough evaluation of the full texts, seven (13–17, 22, 23) articles were excluded, resulting in seven articles remaining (Figure 1).

### 3.2 Study characteristics

Seven studies (13–17, 22, 23) involving 100,487 participants including 23,921 KC patients, were included. All studies were published in 2012 or later. The mean age of participants ranged from 24 to 80. Five of these studies were large population-based studies conducted in the US (16), Denmark (22), Taiwan (13), China (23), and Germany (17). Also, two case-control studies with sample sizes of 103 and 124, were conducted in Greece (14) and Turkey (15), respectively. International Classification of Diseases (ICD) diagnosis codes (13, 16, 23), PHQ-9 (14, 17), BDI (15), and interview (23) were used for depression assessment.

Overall, three studies found a significant association between depression and KC (14, 15, 22). Conversely, Lin et al. (13) reported that depression is a protective factor against developing KC, showing a 42% reduced odds ratio of KC. Also, Xu et al. (23) and Woodward et al. (16) found no significant association between KC and depression.

The main limitation of the studies was the lack of consideration for the index time, which refers to the date of initial KC diagnosis. Only two studies reported the index time (13, 16). Also, in the Bak-Nielsen et al. (22) study, although the index time was not specified, subsequent analysis revealed 108% higher odds of depression compared to controls when accounting for the index time.

### 3.3 ROB

All studies showed low ROB score (Table 1).

### 3.4 Synthesis of results

#### 3.4.1 Risk of depression

**Figure 2:**
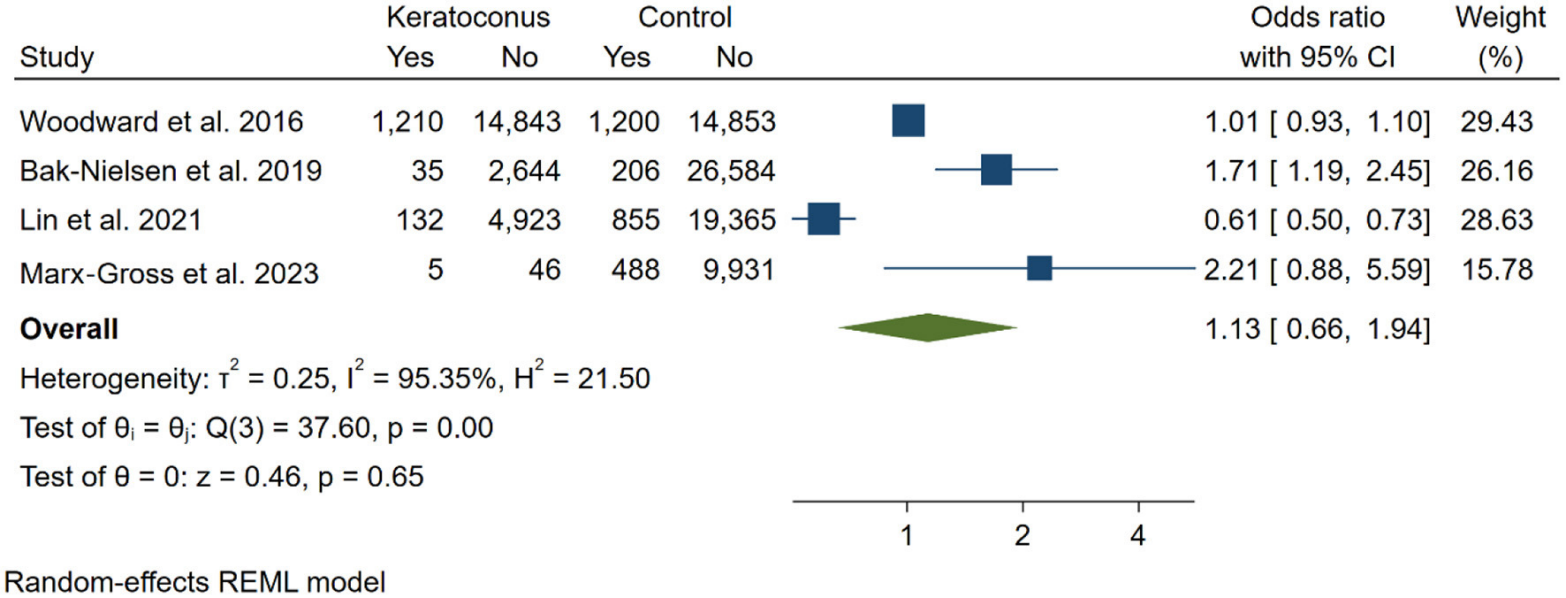
Meta-analysis for the prevalence of depression in all included studies.

To evaluate the risk of depression in individuals with KC compared to control groups, four studies (13, 16, 17, 22) were included in the analysis. A total of 23,838 KC participants and 73,482 controls were analyzed, revealing no significant increase in the risk of depression among KC participants compared to the control group [OR: 1.13 [0.66, 1.94]; *I*^2^: 95.35%; Figure 2]. We performed a sensitivity analysis to assess the individual impact of each study on the odds ratio (OR), which is the primary outcome in our statistical model. This analysis involved systematically removing one study at a time. The exclusion of the study by Bak-Nielsen et al. (22) had a notably greater effect on the overall effect size estimation compared to the other studies [OR = 0.89 [0.42, 1.35], *p* < 0.001].

**Figure 3:**
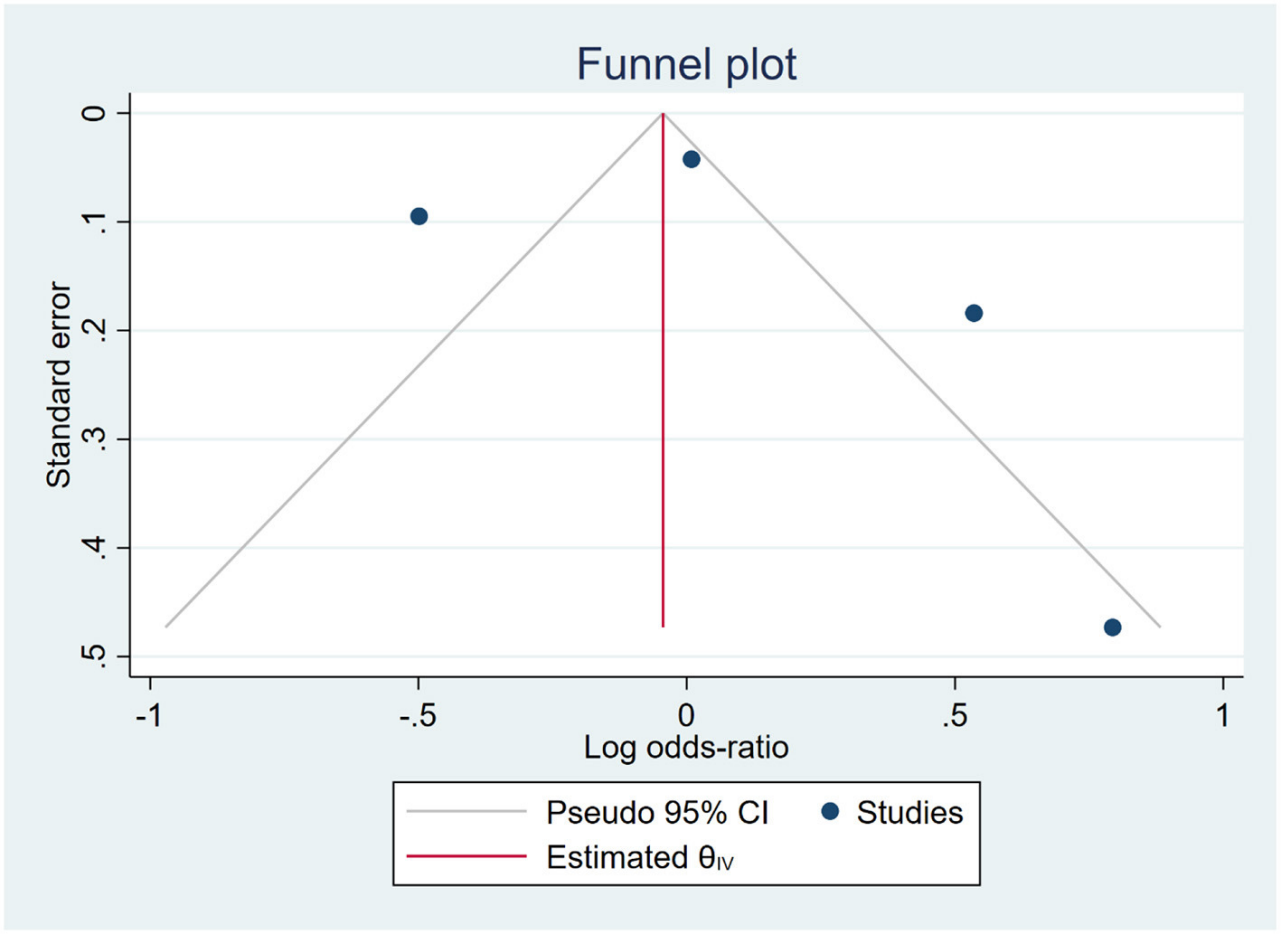
Funnel plot of all included studies for the prevalence of depression.

The funnel plot exhibited an asymmetric distribution of the data, suggesting a potential presence of publication bias. However, this finding was inconsistent with the results of Egger's and Begg's tests, which indicated a low risk of publication bias (*p* = 0.019 and *p* > 0.99, respectively), as shown in Figure 3. Consequently, we applied the trim-and-fill method, which provided evidence of publication bias with the addition of one more study [OR: 1.036 [0.473, 1.599]].

A meta-regression analysis was performed to evaluate the overall influence of the publication year, total population, average age, and percentage of males on the pooled effect size, none of which were found to be significant (Table 2).

**Table 2:** Meta regression based on desired variables.

**Variables**	**Coefficient**	**Standard error**	**95% confidence interval**	***P*-value**
Published year	0.036	0.135	−0.228, 0.301	0.788
Total population	−0.029	0.020	−0.069, 0.011	0.150
Mean age	0.067	0.060	−0.051, 0.183	0.265
Male (%)	−0.768	0.464	−1.677, 0.140	0.097

#### 3.4.2 Depressive score

**Figure 4:**
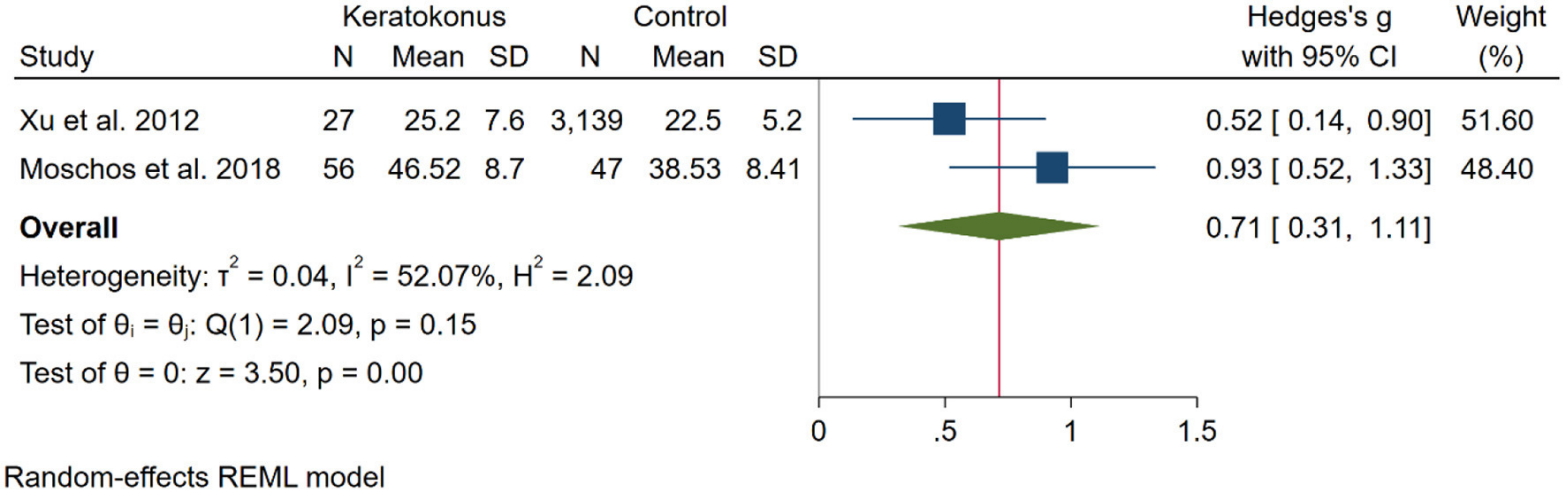
Meta-analysis for depression score of all included studies.

To compare the depression score between KC and control participants two studies (14, 23) were included. In total 83 KC and 3,186 control from these two studies were included in the analysis that showed higher depression scores in KC participants [SMD [95% CI]: 0.71 [0.31, 1.11]; *p* < 0.01; *I*^2^: 52.7%; Figure 4].

## 4 Discussion

Depression significantly influences treatment adherence and outcomes in patients with chronic diseases (24), thereby potentially hindering the course of KC progression and compromising the effectiveness of treatment modalities. In our systematic review and meta-analysis, we found a higher prevalence of depression among KC patients; however, this association was not statistically significant. Furthermore, we observed higher depression scores in patients with KC, indicating a more notable psychological burden within this group of patients.

Several studies have investigated the association between depression and KC and indicated a non-significant association between depression and KC (14, 15, 17, 22). A study by Alfardan et al. (12) showed that patients with KC tend to experience more psychiatric issues, particularly depression, with 56.1% of the 57 patients diagnosed with depression. Al-Dairi et al. (25) discovered that depression is significantly prevalent among KC patients, with a prevalence rate of 40.6%. Notably, this link persists regardless of disease severity or socio-demographic factors.

The assertion that patients with depression demonstrate a protective effect against KC is not adequately supported, as only one study by Lin et al. (13) has explored this association. Lin et al. findings contradict previous studies in the literature due to their reliance on health records without direct verification, potentially leading to diagnosis inaccuracies. Moreover, the study's methodology raises doubts about whether depression genuinely confers protection against KC. Important factors such as eye rubbing and family medical history were not thoroughly examined, and the focus on older patients overlooks potential risk factors in younger individuals. Additionally, cultural stigma in some Asian communities often leads to underreporting of depressive symptoms, which may influence the observed association between depression and KC (26). Consequently, while the study hints at a connection between depression and KC, further research is necessary to confirm this link, by considering these methodological limitations.

Several studies found no significant link between depression and KC (16, 23). In a study by Bak-Nielsen et al. (22), there was no significant difference in the depression prevalence observed between KC patients and the control group. However, after the index time, the prevalence of depression was estimated to be higher among KC patients. Additionally, due to potential misclassification of KC and associated conditions, challenges in differentiating KC from post-refractive surgery ectasia, incomplete coverage of ophthalmological healthcare data in the registry, and delays in diagnosis, their findings may not accurately reflect the expected outcomes. In another study, Jonas et al. (26) found no significant association between several ocular diseases such as KC, and depression prevalence; however, this association was marginally significant in patients with lower visual acuity (VA).

The diversity in depression prevalence among individuals with KC can be attributed to differences in sample sizes, study designs, depression tools, chronicity of KC and methodologies. Smaller sample sizes or less robust study designs may overlook certain aspects of depression prevalence or fail to capture its full extent. Genetic and environmental factors in KC development indirectly influence depression prevalence, as indicated by Gordon-Shaag et al. (27). Genetic predispositions and environmental stressors linked to KC may contribute to mental health challenges, including depression. Sociodemographic factors like age, gender, and location significantly affect KC prevalence and characteristics (22). Furthermore, cultural disparities and healthcare access might influence how depression is expressed and reported among KC patients in different regions or demographic groups. Additionally, diverse diagnostic criteria and tools utilized for KC identification, such as corneal pachymetry, tomography, and topography, can influence reported prevalence rates and associated risk factors (28).

Moreover, differences in KC chronicity and severity may be another source of variation in depression scores. Al-dairy et al. (25) found a significant association between depression and KC regardless KC severity. Bak-Nielsen et al. (22) in Danish national registries, found 108% higher odds of depression compared to controls after the consideration of index time as the time of first KC diagnosis for the KC group and time of matching for the control group; whereas the relationship of KC and depression was significant before index time consideration. This result highlighted the impact of the chronicity of KC on depression. In contrast, another study by Alfardan et al. (12) investigated the chronology of psychiatric illness and KC. They found that 51% of psychiatric illnesses had been diagnosed before KC development, suggesting higher susceptibility of individuals with psychiatric illnesses to KC rather than a causal relationship between them. Further prospective studies with more vigorous methodologies are required to help us understand the relationship of psychiatric illnesses and KC.

The scores obtained in depression assessments among KC patients can vary. In our systematic review and meta-analysis, we found higher depression scores in KC patients. In another study by Moschos et al. (14), 12.5% of KC patients did not suffer from depression according to the PHQ-9 score, while 46.4% encountered mild, 28.6% moderate, and 12.5% severe depressive symptoms. These varying scores reflect the spectrum of depressive symptoms observed in KC patients and the importance of using validated assessment tools to evaluate depression in this population. Based on Durakovic et al.'s (7) research, deteriorating mental health scores were associated with reduced visual acuity in both the better and worse eyes, heightened ocular asymmetry, and worsening disease severity. Mental health effects were frequently found to exceed those related to changes in visual acuity. However, with time, mental wellbeing tended to improve, indicating a potential stabilization of the disease and increased acceptance by patients (29).

We believe that the variation in depression scores among individuals with KC arises from several factors. The severity of the condition, varying from mild to severe, significantly influences the emotional distress experienced by the individual, particularly if their vision impairment is notable (30). The coping mechanisms employed and the level of support received from social circles and family networks also exert a substantial impact. Those with effective stress management techniques and robust support networks typically exhibit lower levels of depression, whereas those lacking support may experience heightened distress (31). Socioeconomic factors such as financial limitations and differences in mental health service access could exacerbate levels of depression (32). Additionally, the presence of concurrent health issues or past adverse experiences can contribute to the complexity of depression management (33). Recognizing these various effects is crucial for developing effective strategies to address depression in individuals with KC.

The bidirectional relationship between depression and KC suggests a complex interplay where each condition may influence the other's development or progression; however, this interaction has not been well-studied. Individuals with depression may exhibit behaviors or habits, such as eye rubbing or neglecting eye care, that could potentially exacerbate KC or contribute to its progression (34). Additionally, the stress and emotional burden associated with depression may compromise immune function or exacerbate inflammation, which is believed to play a role in the pathogenesis of KC (35). KC can potentially impact the quality of life related to vision, with social and physical impairments. Fan et al. conducted a qualitative study and revealed that patients with KC, reported that the visual symptoms they experienced had a profound effect on their education and early career. Consequently, it resulted in their disengagement in school and restricted career opportunities. Moreover, the ability to relish life was also a factor, as they had to reduce their participation in activities and hobbies and experienced emotional distress from lost confidence and the restrictions imposed on their travel. The vision and emotional state of the individuals, along with financial problems, had a negative impact on their relationships and driving contributing to frustration and susceptibility to depression (36). Similarly, another qualitative study by Fournie et al. showed that KC patients, report sense of fear, worry and anxiety due to their condition, such as concerns for visual disturbance, car accidents due to impaired sight, and anxiety about potential eye surgery (37).

In another study, Steinberg et al. revealed that anxiety about the uncertain future effects of vision loss can significantly impact a patient's mental health (38). Consequently, longitudinal studies that investigate the onset of ocular disease and the development of depression as a consequence are crucial for understanding the bidirectional association between ocular disease and mental wellbeing and developing particular strategies to improve psychological outcomes in these patients (39).

We hypothesize that the physical manifestations and visual impairment linked to KC can significantly impact an individual's psychological wellbeing, potentially leading to or worsening depressive symptoms. Visual challenges, including difficulty in daily activities, social interactions, and occupational tasks, may contribute to feelings of frustration, isolation, and low self-esteem, all characteristic of depression. Additionally, the chronic nature of both depression and KC can create a cycle where each condition exacerbates the other. For instance, stress and anxiety resulting from KC progression may worsen depressive symptoms, while individuals with depression may struggle to adhere to KC treatment, impacting their eye health and potentially worsening KC symptoms (29, 39). Recognizing the bidirectional relationship between depression and KC is essential for providing comprehensive care to affected individuals. By addressing both the physical and psychological aspects of these conditions, healthcare providers can potentially enhance patient support and improve overall wellbeing.

A notable strength of our study lies in its innovative nature as it is the first systematic review performed in this particular field. Using a meta-analysis methodology, we synthesized data from several high-quality studies to offer a precise overview of the correlation between ocular disease and mental health. Including comprehensive studies improves the reliability and validity of our findings and enhances the robustness of our conclusions.

The study has several limitations. First, the inclusion of studies with retrospective designs, may introduce biases that compromise the reliability of our findings. Secondly, limited sample sizes in several studies restrict the generalizability of our findings to larger populations. Thirdly, the absence of all ethnic groups in the included studies may limit the applicability of the findings to diverse populations. Moreover, the high heterogeneity among studies could influence pooled results and affect result interpretation. Despite our meta-regression analysis not identifying any significant determinants of heterogeneity, several potential sources may be contributing to this variability. These include differences in study design, participants' characteristics, study quality, and publication bias. Addressing the issue of heterogeneity is a multi-faceted task, which involves the investigation of new variables, as well as the improvement of study methodologies. Lastly, potential publication bias underscores the need for precise interpretation, as it may lead to an incorrect estimation of depression prevalence rates.

Future research should focus on larger-scale studies and across diverse settings and populations to improve precision in elucidating the mechanisms underlying the relationship between KC onset and depression. Moreover, they should be directed at recognizing and controlling possible heterogeneity sources to improve the strength and applicability of meta-analytic results. Prospective cohort studies are recommended for capturing longitudinal data and identifying causal relationships. Additionally, evaluating interventions to mitigate the impact of ocular disease on mental wellbeing is essential for informing targeted strategies.

## 5 Conclusion

In conclusion, while the analysis of depression scores suggested significantly higher levels in KC patients compared to controls, the meta-analysis of depression rates found no increased risk of depression in KC patients. These contradictory findings highlight the need for further research to clarify the relationship between keratoconus and depression. Larger, well-designed studies with standardized assessment methods are necessary to provide a more definitive conclusion. Nonetheless, the review underscores the importance of considering mental health in the management of KC patients to ensure comprehensive and holistic care.

## Data Availability

The original contributions presented in the study are included in the article/Supplementary material, further inquiries can be directed to the corresponding authors.
