# Supplementary material for: Depression among keratoconus patients: a systematic review and meta-analysis
Source: Front Public Health. 2024 Nov 22;12:1477411. doi: 10.3389/fpubh.2024.1477411 (PMC11621007; doi:10.3389/fpubh.2024.1477411)
Supplement: Supplementary file 1 [file Data_Sheet_1.docx]

**Databases**

1. **PubMed: 39 results**

(("Keratoconus"[Mesh]) OR (Keratoconus*[Title/Abstract])) OR ("Pellucid Marginal Degeneration"[Title/Abstract])

(((Depression*[Title/Abstract]) OR (depress*[Title/Abstract])) OR ("depressive disorder*"[Title/Abstract]))

Final: #1 AND #2

1. **Scopus: 78 results**

TITLE-ABS-KEY(“Pellucid Marginal Degeneration”) OR TITLE-ABS-KEY(Keratoconus*)

TITLE-ABS-KEY(Depression*) OR TITLE-ABS-KEY(depress*) OR TITLE-ABS-KEY(“depressive disorder*”)

Final: #1 AND #2

1. **Web of sciences: 34 results**

TS=(“Pellucid Marginal Degeneration” OR Keratoconus*)

TS=( Depression* OR depress* OR “depressive disorder*”)

Final: #1 AND #2

1. **PsycINFO: 1 results**

TI “Pellucid Marginal Degeneration” OR TI Keratoconus* OR AB “Pellucid Marginal Degeneration” OR AB Keratoconus*

TI Depression* OR TI depress* OR TI “depressive disorder*” OR AB Depression* OR AB depress* OR AB “depressive disorder*”

Final: #1 AND #2

1. **CINAHL complete: 7 results**

TI “Pellucid Marginal Degeneration” OR TI Keratoconus* OR AB “Pellucid Marginal Degeneration” OR AB Keratoconus*

TI Depression* OR TI depress* OR TI “depressive disorder*” OR AB Depression* OR AB depress* OR AB “depressive disorder*”

Final: #1 AND #2
